# Circulating soluble CD36 as a novel biomarker for progression and prognosis of HBV-related liver diseases

**DOI:** 10.3389/fmicb.2022.1039614

**Published:** 2022-11-02

**Authors:** Chunxian Cai, Anhua Xiao, Xiaoqing Luo, Enze Zheng, Yiyu Li, Yu Lei, Shan Zhong, Yaxi Chen, Ping Yang, Zhurong Tang, Zhi Zhou

**Affiliations:** ^1^Department of Infectious Diseases, The Second Affiliated Hospital, Chongqing Medical University, Chongqing, China; ^2^Centre for Lipid Research, Key Laboratory of Molecular Biology for Infectious Diseases, Ministry of Education, Chongqing Medical University, Chongqing, China; ^3^Institute of Life Sciences, Chongqing Medical University, Chongqing, China

**Keywords:** sCD36, biomarker, HBV-related liver diseases, cirrhosis, liver failure

## Abstract

**Background:**

Our previous study suggested CD36 may be a positive regulator of hepatitis B virus (HBV) replication *in vitro*. Therefore, the present study aimed to investigate whether circulating soluble CD36 (sCD36) could serve as a diagnostic and prognostic biomarker for HBV-related liver diseases based on the clinic collected data.

**Methods:**

A total of 282 subjects were divided into healthy controls (HC, *n* = 47), chronic hepatitis B (CHB, *n* = 68), HBV-related liver cirrhosis (HBV-LC, *n* = 167). Soluble CD36 in plasma was measured by ELISA, and monocyte or platelet CD36 expression was determined by flow cytometry.

**Results:**

There was a step-wise increase of sCD36 with the progression of chronic HBV infection, and it was the highest in the HBV- LC group with liver failure (1.50, IQR:1.04–2.00) as compared with HC (0.38, IQR:0.27–0.38), CHB (0.75, IQR:0.40–1.13), and HBV-LC without liver failure (1.02, IQR,0.61–1.35) group. Circulating sCD36 was not correlated with serum HBV DNA levels, but correlated with liver function parameters. Additionally, ROC analysis confirmed sCD36 could be used to predict liver failure for HBV-LC patients, which yielded an AUC of 0.775 with 71.0% sensitivity and 72.2% specificity. Multivariate logistic regression analysis revealed sCD36 is an independent risk factor in predicting liver failure. Moreover, plasma sCD36 in HBV-LC patients was significantly correlated with prognostic indices, including MELD, MELD-Na and CHILD-PUGH scores. On the other hand, CD36 expression on monocytes or platelets was positively correlated with plasma sCD36 levels, whereas they were not strongly associated with the disease severity.

**Conclusion:**

Circulating sCD36 could be used as a novel noninvasive biomarker for predicting liver failure and prognosis in chronic HBV infected patients.

## Introduction

Hepatitis B virus (HBV) infection is a growing global public health problem with high morbidity and mortality. HBV belongs to a small DNA virus that predominately infects hepatocytes, thus leading to hepatitis, liver cirrhosis and liver cancer (Fattovich et al., 2008). Liver failure can occur in any stage of HBV infection, and is clinically manifested as hepatic encephalopathy, ascites, hepatorenal syndrome, coagulation dysfunction and jaundice, which finally results in multiple organ failure and high mortality (Seto et al., 2012). To control HBV-related diseases, diagnosis the progression of chronic HBV infection with noninvasive biomarkers would be helpful and low-cost. While discovering these biomarkers often benefit from clarifying mechanisms involved in progression of HBV infection, which, however, remain incompletely revealed yet.

Chronic HBV infection is a dynamic process which reflects the interaction between virus replication and the host immune response. Noteworthy, monocytes and macrophages, which play important roles in progression of HBV infection, are involved in liver fibrosis and development of cirrhosis including liver failure (Zimmermann et al., 2010; Tacke, 2012; Kazankov et al., 2014; Tacke and Zimmermann, 2014; Gronbaek et al., 2016). Besides, systemic inflammation is also a striking feature of acute-on-chronic liver failure. For example, proinflammatory molecules such as IL-6, TNF-α, and IL-8 were elevated in HBV patients with acute-on-chronic liver failure (Claria et al., 2016). Additionally, HBV is recognized as a metabolovirus for its close connection with the host metabolism (Shlomai and Shaul, 2008). Studies have found that the serum lipid profile is altered in patients with HBV infection (Buechler and Aslanidis, 1865; Chen et al., 2013), and there is an increasing level of fatty acid in serum along with the progression of HBV infection (Schoeman et al., 2016). Similar studies have found that total fatty acids were substantially increased in the HBV-replicating cell lines compared with the original cell lines, which indicates the fatty acid biosynthesis is enhanced by HBV infection (Zhang et al., 2013; Li H. et al., 2015).

Fatty acid translocase (FAT/CD36) is a multifunctional receptor expressed in various tissues and has a variety of tissue-specific functions. CD36 on the surface of muscle and fat tissue is associated with uptake of long-chain fatty acids，and contributes to obesity-induced insulin resistance (Su and Abumrad, 2009); CD36 on the surface of macrophage cells mediates sterile inflammation caused by production of endogenous molecules, and phagocytosis of pathogens (Baranova et al., 2008; Stewart et al., 2010); CD36 overexpression on the surface of hepatocytes is linked to development of nonalcoholic fatty liver disease (NAFLD), including fatty liver, steatohepatitis, and cirrhosis (Zhao et al., 2018); and increased CD36 expression on the surface of platelets, monocytes and macrophages is involved in the pathogenesis of type 2 diabetes (Sampson et al., 2003; Liani et al., 2012). Previous studies have identified a soluble form of CD36 (sCD36) in circulation as a novel biomarker for NAFLD, type 2 diabetes mellitus and atherosclerosis (Handberg et al., 2012; Liani et al., 2012; Heeboll et al., 2017). Whether sCD36 could be a biomarker of HBV-related diseases is still unknown to our knowledge.

Our previous studies have found a novel link between CD36 and HBV replication *in vitro*, which was mediated by Src kinase pathway (Huang et al., 2016, 2017). Moreover, CD36 is a co-receptor of hepatitis C virus (HCV) E1 protein, mediating enhanced HCV entry and replication in hepatic cells (Cheng et al., 2016). These studies suggested there might exist a close link between CD36 and hepatic viruses. Therefore, this study aimed to evaluated the potential of sCD36 in diagnosing the progression and prognosis of HBV-related liver diseases.

## Materials and methods

### Patients and study design

A total of 235 patients, including 68 cases of chronic hepatitis B (CHB), 94 cases of HBV-related cirrhosis without liver failure (HBV-LC, NO LF) and 73 cases of HBV-LC with liver failure (HBV-LC, LF) were collected from the Second Affiliated Hospital of Chongqing Medical University. CHB was diagnosed based on patients with hepatitis B surface antigen and (or) HBV-DNA positive for more than 6 months. LC was diagnosed based on ultrasound and CT examination. LF was diagnosed by International Normalized Ratio (INR) >1.5 or prothrombin activity (PTA) <40%. Healthy individuals (*n* = 47) examined in the physical examination center of the same hospital were included as the healthy control (HC).

In detail, hospitalized patients were screened for chronic HBV infection as defined by hepatitis B surface antigen and (or) HBV-DNA positive for at least 6 months with impaired liver function (Chinese Society of Infectious Diseases, C.M.A., and Chinese Society of Hepatology, C.M.A, 2019). The following exclusion criteria for the hospitalized patients were applied: patients who received immunomodulatory therapy in the past 12 months; with metabolic diseases except obesity (including diabetes, non-alcoholic fatty liver disease, hyperlipemia and atherosclerosis); with evidence of carcinoma; with human immunodeficiency virus infection; with evidence of contaminant liver diseases; or with evidence of systemic sclerosis.

HBV-related liver diseases included chronic hepatitis B (CHB), cirrhosis and liver failure. CHB patients could be divided into mild group and moderate-to-severe group as referred to the Guideline on prevention and treatment of chronic hepatitis B in China (2021). In detail, the mild group included the CHB patients who had no obvious symptoms, or those had certain symptoms or signs but with only 1–2 mild abnormal biochemical index. The severe group contained the CHB patients who had obvious or continued hepatitis symptoms, such as fatigue, anorexia, abdominal distension, loose stool and others symptoms. And they may be accompanied with hepatic face, liver palms, spider angioma or liver splenomegaly that portal hypertension or other origin have been rule out. Their laboratory tests showed repeated or continuous increase in serum ALT, decreased albumin or abnormal A/G ratio, and significantly increased gamma globulin. A severe diagnosis can be made if one of the three tests meets the criteria: albumin ≤32 g/ L, bilirubin >85.5 μmol/l and prothrombin activity was between 60 and 40%. The CHB patients whose symptoms, signs, and laboratory tests range from mild to severe were included in the moderate group. Cirrhosis could be divided into compensated cirrhosis and decompensated cirrhosis (usually leading to liver failure) which was defined by the acute development of large ascites, hepatic encephalopathy (HE), gastrointestinal bleeding (GI bleeding), bacterial infection, or any combination of these symptoms. Liver failure occurred in cirrhosis can be divided into acute-on-chronic liver failure (ACLF) and chronic liver failure (CLF) according to the course of the disease. ACLF was characterized by acute deterioration of liver function which caused the failure of the liver and other extrahepatic organs (Bernal et al., 2015). However, CLF was characterized by slow progressive deterioration of liver function, causing chronic decompensation and mainly manifested by recurrent ascites and/or hepatic encephalopathy.

Blood samples were obtained from all study individuals at enrollment and we collected data including laboratory measurements by the automatic blood or biochemical analyzers in the hospital. The HBV-DNA was determined *via* quantitative real-time PCR, and the hepatitis B surface antigen was detected by ELISA. Events that may be potential precipitating factors of liver failure (including ascites, bacterial infection, GI bleeding and HE) were also recorded. Patient characteristics and platelet and monocyte sample data were summarized in Table 1.

**Table 1:** Baseline characteristics of all enrolled subjects.

Parameter	HC (N = 47)	CHB (N = 68)	HBV-related cirrhosis (HBV-LC)		p value
			NO LF (N = 94)	LF (N = 73)	
BMI	22.19 (19.2–23.7)	22.8 (19.8–25.5)	23.1 (20.8–25.1)	22.2 (20.3–24.0)	0.439
Age (years)	32 (28–36)	34 (26–38)	49 (43–55) ^*^	48 (42–54) ^*^	<0.001
Male (N, %)	21 (44.7)	55 (80.9) ^*^	67 (71.3) ^*^	55 (75.3) ^*^	0.376
HE (N, %)	0	0	1 (1.1)	4 (5.5)	
Ascites (N,%)	0	3 (4.4)	35 (37.2)	48 (65.8)	<0.001
Bacterial infection (N, %)	0	9 (13.2)	39 (41.5)	51 (69.9)	<0.001
GI bleeding (N, %)	0	0	8 (8.5)	7 (9.6)	
Laboratory data					
PLT (^*^10^9/L)	205 (160–227)	179 (129–216)^*^	77 (50–108)^*^	54 (36–85)^*^	<0.001
TBil (mg/dl)	0.5 (0.2–0.7)	1.2 (0.7–3.3)^*^	1.3 (0.7–2.6)^*^	5.2 (1.6–13.8)^*^	<0.001
ALT (U/L)	19 (12–24)	136 (58–289)^*^	32 (21–78)^*^	41 (21–109)^*^	<0.001
AST (U/L)	18 (14–22)	57 (36–107)^*^	41 (28–81)^*^	57 (36–131)^*^	0.08
Albumin (g/L)	47.1 (44.7–49.1)	38.9 (35.2–42.1)^*^	34.2 (30.5–38.3)^*^	30.1 (21.7–31.7)^*^	<0.001
HBV-DNA (log IU/ml)		2.0 (0–5.5)	2.0 (0.0–5.5)	3.1 (0–5.1)	<0.001
INR		1.1 (1.0–1.1)	1.3 (1.1–1.4)	1.9 (1.7–2.2)	<0.001
PTA		89 (79–101)	71 (61–81)	42 (35–49)	<0.001
Cr (mg/dl)	0.7 (0.6–0.9)	0.8 (0.6–0.8)	0.8 (0.7–0.9)	0.7 (0.6–0.8)	0.026
Serum sodium (mmol/L)		138.6 (137.1–139.7)	137.9 (135.6–139.2)	135.3 (132.5–137.9)	<0.001

Data are expressed as median (interquartile range) or count (percentage), Statistical analysis was calculated using the man-whitney U test between two groups and the Kruskal-Wallis test among three groups. *p* value shows the difference among the HC group, HBV-LC (NO LF) group and HBV-LC (LF) group. *p* < 0.05 is considered significantly statistical difference. HC: healthy controls; CHB: chronic hepatitis B; HBV-LC (NO LF): HBV-related cirrhosis without liver failure; HBV-LC (LF): HBV-related cirrhosis with liver failure; BMI: body mass index; HE: hepatic encephalopathy; GI bleeding: gastrointestinal bleeding; PLT: platelets; ALT: alanine aminotransferase; AST: aspartate aminotransferase; TBil: total bilirubin; INR: international normalized ratio; PTA: prothrombin activity; Cr: creatinine.

* *p* < 0.05 compared with HC group.

The study complied with the World Medical Association Declaration of Helsinki. Written informed consents were obtained from patients or their legal surrogates before enrollment. The study was approved by the Committee on Ethics of the Second Affiliated Hospital of Chongqing Medical University.

### Detection of plasma sCD36

The plasma concentrations of sCD36 were determined in samples that had been frozen at −80°C using a sandwich enzyme-linked immunosorbent assay (ELISA) kit (E0674h, EIAab Science Inc., Wuhan, China) and detected by a HIMFD microplate reader. Control samples and CD36 standard serum with concentrations that ranged from 0.156 to 10 ng/ml were included in each run. The inter-assay coefficient of variation was less than 7.1% and intra-assay coefficient of variation was less than 3.2%. The limit of detection (lowest standard) was 0.156 ng/ml.

### Detection of CD36 expression on monocytes and platelets

Quantification of CD36 expression on monocytes or platelets was done by flow cytometry. At first whole-blood samples with heparin were immediately moved to the lab and processed. Both peripheral blood mononuclear cells and platelets were isolated using a mouse peripheral blood mononuclear cell isolation kit (P5230, Solarbio Science & Technology Co., Beijing, China). Allophycocyanin (APC) conjugated CD14 antibody (BD Biosciences, New Jersey, United States) was used to identify monocytes and phycoerythrin (PE) conjugated CD36 antibody (Thermo Fisher Scientific, Massachusetts, United States) was used to identify CD36 on the monocytes and platelets surface, with CD36 single dye tube, CD14 single dye tube and blank tube served as controls. The surface intensity of antigens on peripheral blood monocytes or platelets was determined using the BD FACS Aria II cytometer (BD Biosciences, United States) and the dot plot (the two-dimensional histogram that show two parameters) was used for evaluating the FACS data.

### Statistical analysis

Quantitative variables were reported as mean and SD if normally contributed, or median and interquartile range if not. Categorical variables were reported as counts or percentages in each category. Correlations of the sCD36 with every other variable were analyzed using the Pearson’s correlation coefficient if normally distributed, and the Spearman’s rank test if not. Kruskal-Wallis test was used for non-normal distribution to compare quantitative variables across multiple groups in the study. Comparison between two groups was assessed by means of t test if normally distributed or Man-Whitney U test if not. Categorical variables were analyzed by chi-square test. Univariate and multivariate logistic regression analysis were performed to identify the main effects associated with HBV-related cirrhosis with liver failure. Receiver-operating characteristic (ROC) curves were plotted to measure the performance of sCD36 for diagnosis of HBV-related liver diseases. In addition, ROC curves were used to determine the cut-off value of sCD36 expression levels with the best sensitivity and specificity in discriminating different stages of HBV-related liver diseases. If the available sample data in one of the groups were very small or incomparable, no statistical comparison was carried out. The significance level for all tests was set at 5%. Statistical analyses were conducted using SPSS software version 21 (SPSS Inc., Chicago, IL, United States).

## Results

### Basic clinical and biochemical data

All patients were divided into three groups at enrollment according to disease status, including CHB group, HBV-related cirrhosis without liver failure named HBV-LC (NO LF) group, and HBV-related cirrhosis with liver failure named HBV-LC (LF) group, and the HC group was used as control, as described in the methods. Body mass index (BMI) was similar among the four groups. The platelet, serum albumin, PTA, and serum sodium levels in HBV-infected patients were lower than those in healthy adults, and gradually decreased with the progression of chronic HBV infection (Table 1, *p* < 0.001). On the contrary, levels of serum total bilirubin (TBil), INR, alanine transaminase (ALT) and aspartic transaminase (AST) were significantly higher in HBV-infected patients than those in healthy adults, and TBil and INR were gradually increased with the progression of chronic HBV infection (Table 1, *p* < 0.001). HBV-DNA were significantly different among the three HBV-infected groups (Table 1, *p* < 0.001). With the progression of chronic HBV infection, the proportion of ascites and bacterial infection gradually increased (Table 1, *p* < 0.001). The distribution of serum creatinine in HBV-infected patients was similar with healthy adults, as shown in Table 1.

### Plasma sCD36 increased with the severity of HBV-infected liver diseases

To examine the role of CD36 in HBV-infected liver diseases, firstly, we compared plasma sCD36 level in the above four groups. There was a step-wise increase of sCD36 with the progression of chronic HBV infection. The sCD36 level in the three HBV-infected groups were significantly higher than that of the healthy control group (0.38, IQR, 0.27–0.38). Compared with the sCD36 level in chronic hepatitis B patients (0.75, IQR, 0.40–1.13), it was higher in HBV-related cirrhosis patients with (1.50, IQR, 1.04–2.00) or without liver failure (1.02, IQR, 0.61–1.35). And the cirrhosis patients with liver failure also had significant higher sCD36 level in comparison with those without.

**Figure 1:**
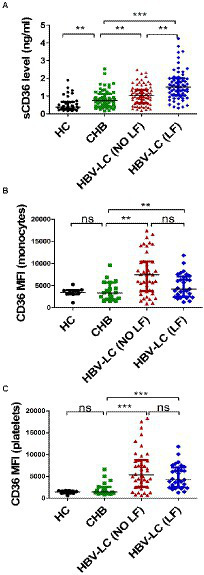
Comparison of circulating CD36 expression among different groups with HBV-related liver diseases. All subjects were divided into four groups at enrollment according to the diseases status, including healthy control (HC) group, chronic hepatitis B (CHB) group, HBV-related cirrhosis without liver failure (HBV-LC, NO LF) group, and HBV-related cirrhosis with liver failure (HBV-LC, LF) group. **(A)** Plasma sCD36 level in HC (*n* = 38), CHB (*n* = 61), HBV-LC (NO LF; *n* = 77), and HBV-LC (LF; *n* = 67) groups. **(B)** CD36 expression on monocytes in HC (*n* = 9), CHB (*n* = 19), HBV-LC (NO LF; *n* = 41), and HBV-LC (LF; *n* = 29) groups. **(C)** CD36 expression on platelets in HC (*n* = 9), CHB (*n* = 19), HBV-LC (NO LF; *n* = 41), and HBV-LC (LF; *n* = 29) groups. Each point represents one participant. Values are described by median (interquartile range). The differences between two groups were analyzed by Man - Whitney U test. ^**^p < 0.01, ^***^p < 0.001, ns, no statistical difference.

The CD36 mean fluorescence intensity (MFI) on monocytes in CHB group was 3,362 (IQR:1871–5,709), which was similar with that of the HC group [2,764 (IQR: 2235–3,220), *p* > 0.05], while it was significantly lower than that of the HBV-LC (NO LF) group [7,450 (IQR: 3757–10,441), *p* < 0.05] and HBV-LC (LF) group [6,303 (IQR: 4738–8,322), *p* < 0.05] (Figure 1A). Whereas, no significant differences were found between the HBV-LC (NO LF) and HBV-LC (LF) group (*p* > 0.05, Figure 1B). Similarly, the CD36 MFI on platelets in the CHB group was 1,408 (IQR: 1108–2,576), which was similar with that in the HC group [1,430 (IQR: 1111–1707), *p* > 0.05], while it was lower than that of the HBV-LC (NO LF) [5,512 (IQR: 2584–9,208), *p* < 0.001] and HBV-LC (LF) group [4,216 (IQR: 2545–7,043), *p* < 0.001]. And no difference between the last two groups were found (*p* > 0.05, Figure 1C).

**Figure 2:**
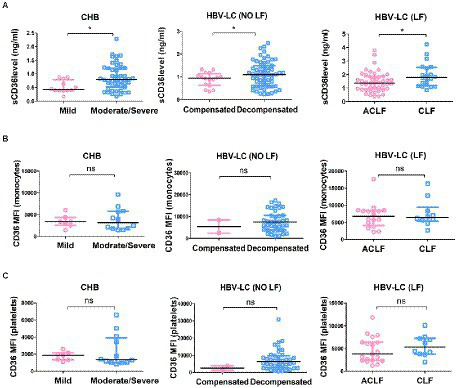
Correlation analysis between plasma CD36 and the severity of HBV-related liver diseases. CHB patients were divided into mild group and moderate-to-severe group, HBV-LC (NO LF) patients were divided into compensated group and decompensated group, and HBV-LC (LF) patients were divided into acute-on-chronic liver failure (ACLF) group and chronic liver failure (CLF) group. **(A)** Plasma sCD36 levels, **(B)** the expression of CD36 on monocytes, and **(C)** platelets in the above groups. ^*^p < 0.05 as determined by Man - Whitney U test for non-parametric data or t test for parametric data. ^*^p < 0.05, ns, no statistical difference.

To further confirm circulating sCD36 increased with the severity of HBV-infected liver diseases, 68 patients with CHB were divided into mild group and moderate-to-severe group, 94 patients with HBV-LC (NO LF) were divided into compensated group and decompensated group, and 73 patients with HBV-LC (LF) were divided into ACLF group and CLF group. The sCD36 levels was significantly higher in moderate-to-severe subgroup than mild subgroup in CHB patients [0.80 (IQR: 0.52–1.21) vs. 0.45 (IQR: 0.39–0.85), *p* < 0.05], and it was also higher in decompensated subgroup than compensated subgroup in HBV-LC (NO LF) patients (0.89 ± 0.31 vs. 1.10 ± 0.58 ng/ml, *p* < 0.05). And higher sCD36 levels was revealed in CLF subgroup than that of ACLF subgroup in HBV-LC (LF) patients [1.37 (IQR: 0.94–1.83) vs. 1.75 (IQR: 1.13–2.52), *p* < 0.05] (Figure 2A). However, monocyte CD36 MFI was similar between mild and moderate-to-severe subgroup (3,461 ± 1,440 vs. 3,937 ± 2,590, *p* > 0.05), as well as that between compensated and decompensated subgroup (5,424 ± 4,248 vs. 7,725 ± 4,770, *p* > 0.05), and so was the ACLF and CLF subgroup [6,172 (IQR: 3653–8,349) vs. 6,439 (IQR: 5380–9,451), *p* > 0.05] (Figure 2B). Similarly, no significant differences were found for the expression of CD36 on platelets between the above subgroups (Figure 2C). Thus, these data indicated that plasma sCD36 is better than monocyte or platelet CD36 in identifying the severity of HBV-infected liver diseases.

### No correlation between plasma sCD36 and serum HBV DNA load

**Figure 3:**
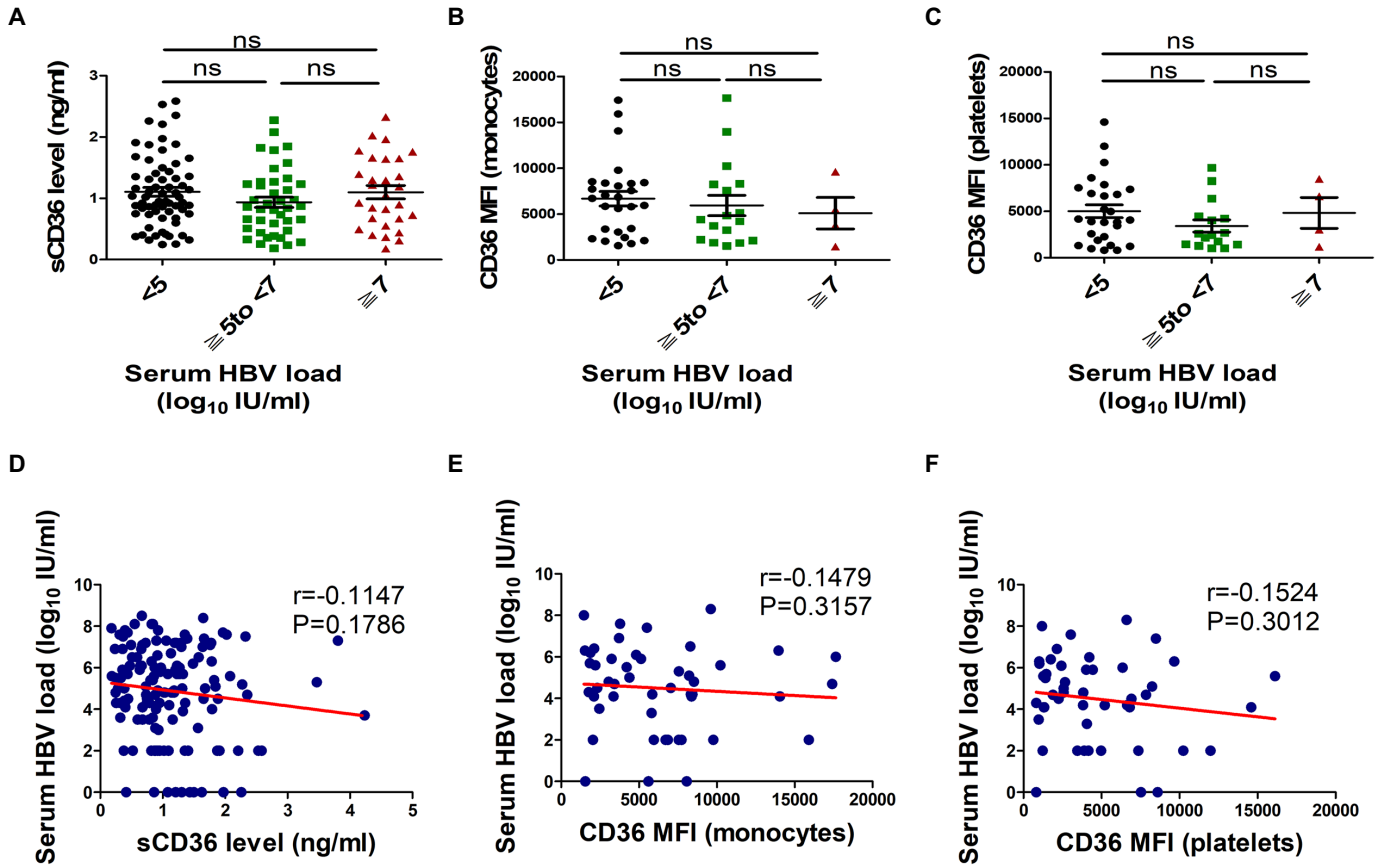
Correlations analysis of plasma sCD36 levels with HBV DNA in HBV-infected patients. Plasma sCD36 level **(A)**, monocyte CD36 expression **(B)** and platelet CD36 expression **(C)** with different viral load (< 5, ≥ 5–7 and ≥ 7 log10 IU/mL); d-f Correlation analysis between HBV DNA levels with plasma sCD36 **(D)**, monocyte CD36 expression **(E)** and platelet CD36 expression **(F)**. The differences between two groups were analyzed by Man - Whitney U test. Correlation analysis were calculated using the Spearman’s rank correlation for non-parametric data. ns, no statistical difference.

Then we explored whether sCD36 correlates with HBV replication in HBV-infected patients. According to serum HBV DNA levels, HBV-infected patients were divided into three subgroups, including the high viral load group (≥ 7 log_10_ IU/mL), moderate viral load group (≥ 5–7 log_10_ IU/mL), and low viral load group (< 5 log_10_ IU/mL). We found that plasma sCD36 levels were similar between these three groups (*p* > 0.05; Figure 3A). The results of CD36 expression on platelets and monocytes were similar with that of sCD36 (Figures 3B,C). In addition, in HBV-infected patients, there was no correlation between HBV DNA levels and sCD36 levels (*r* = −0.1147, *p* > 0.05, Figure 3D), nor between HBV DNA and CD36 expression on monocytes (*r* = −0.1479, *p* > 0.05, Figure 3E) or platelets (*r* = −0.1524, *p* > 0.05, Figure 3F).

### Correlation between plasma sCD36 levels and liver function parameters

To evaluate the association between sCD36 and the clinic parameters of HBV-infected patients, we used chi-square test. No obvious correlation was found between sCD36 and age, sex, AST, and ALT (Supplementary Table 1). Whereas, we found sCD36 was positively correlated with serum TBil, and it was negatively correlated with serum albumin, indicating sCD36 was in association with liver function (Supplementary Table 1). In accordance with this, sCD36 was positively correlated with INR, and it was negatively correlated with PTA, indicating sCD36 was in association with coagulation function (Supplementary Table 1). Additionally, increased sCD36 levels were significantly correlated with platelets, ascites and bacterial infection (*p* < 0.05, Supplementary Table 1).

We then analyzed the correlation between plasma sCD36 and aspartate transaminase-to-platelet ratio index (APRI), and fibrosis-4 score (FIB-4; N = 61), both factors are recognized as noninvasive tests predicting liver fibrosis in CHB patients. Plasma sCD36 level was positively related to APRI (*p* < 0.05, R = 0.284) and FIB-4 (*p* < 0.05, R = 0.259) in CHB patients, though the correlation was weak (Supplementary Figure 1 A). We also analyzed the correlation between monocyte/platelet CD36 expression and APRI, FIB-4 in CHB patients (N = 19). However, there was no significant correlation between monocyte CD36 expression and APRI (*r* = 0.243, *p* > 0.05) or FIB-4 (*r* = 0.277, *p* > 0.05), and no significant correlation was found between platelet CD36 expression and APRI (*r* = 0.037, *p* > 0.05) or FIB-4 (*r* = 0.436, *p* > 0.05) in CHB patients (Supplementary Figures 1 B,C).

### Plasma sCD36 could be used as a strong predictor for liver failure in HBV-LC patients

**Figure 4:**
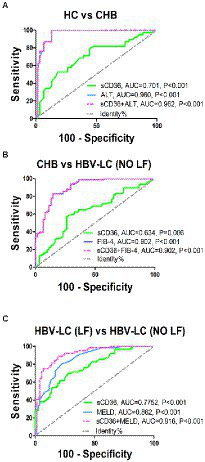
Discriminate validity of plasma sCD36 for HBV-related liver diseases with different stages. **(A)** ROC curves of plasma sCD36, ALT and their combination for discriminating CHB from HCs; **(B)** ROC curves of plasma sCD36, FIB-4 and their combination for discriminating HBV-LC (NO LF) from CHB; **(C)** ROC curves of plasma sCD36, MELD and their combination for discriminating HBV-LC (NO LF) from HBV-LC (NO LF); The Youden index was used to maximize the potential effectiveness of the biomarkers. AUC: area under the curve.

We evaluated the potential of plasma sCD36 in distinguishing different progression stage of HBV-associated liver disease. The ROC curve analysis indicated plasma sCD36 can discriminate CHB from HC with an area under the ROC curve (AUROC) of 0.701 (95%CI 0.592–0.810, cutoff = 0.73 ng/ml), but the discriminate validity was significantly lower than ALT with AUROC of 0.960 (95%CI 0.922–0.998, cutoff = 44 U/l, Figure 4A). Additionally, plasma sCD36 can be used to distinguish HBV-LC (NO LF) from CHB in some extent, and the AUROC was 0.634 (95%CI 0.540–0.728, cutoff = 0.93 ng/ml) with 58.4% sensitivity and 73.8% specificity (Figure 4B). We then explored the potential of sCD36 in predicting liver failure in HBV-LC patients. ROC analysis showed sCD36 exhibited favorable predictive value for liver failure in HBV-LC patients, as it yielded an AUROC of 0.777 (95%CI 0.700–0.855, cutoff = 1.24 ng/ml) with 71.0% sensitivity and 72.2% specificity (Figure 4C). In addition, the combination of sCD36 and MELD exhibited a significantly higher AUROC compared with sCD36 or MELD alone.

Univariate and multivariate regression analyses also revealed sCD36 could be used as a strong predictor for liver failure (Table 2). In accordance with previous studies, univariate analyses showed serum AST, serum albumin, serum TBil, serum cholinesterase, platelets, serum sodium, ascites and bacterial infection were significant risk factors of liver failure (Table 2). Upon multivariate analysis, elevated sCD36 was proved to be an effective predictor for liver failure (odds ratio [95% CI], 3.166 [1.617–6.199], *p* < 0.05), which was independent of platelets, TBil, and ascites (Table 2). Altogether, these results suggest a high potential of sCD36 in predicting liver failure.

**Table 2:** Univariate and multivariate logistic regression analyses of liver failure in patients with HBV-LC patients.

Variable	Univariate analysis		Multivariate analysis	
	OR (95% CI)	*p*	OR (95% CI)	*p*
Age	0.759 (0.485–1.188)	0.228		
Male gender	1.332 (0.641–2.768)	0.443		
sCD36	2.905 (1.672–5.049)	<0.001	3.166 (1.617–6.199)	0.001
ALT	1.147 (0.787–1.616)	0.474		
AST	1.532 (1.038–2.262)	0.032		
Albumin	2.915 (1.705–4.985)	<0.001		
TBil	2.505 (1.676–3.746)	<0.001	3.285 (1.933–5.584)	<0.001
Serum cholinesterase	1.555 (1.017–2.378)	0.041		
Platelet	2.288 (1.425–3.672)	0.001	3.353 (1.800–6.244)	<0.001
Serum sodium	3.757 (1.744–8.094)	0.001		
HBV-DNA	1.070 (0.708–1.616)	0.748		
Ascites	2.282 (1.456–3.578)	<0.001	2.375 (1.344–4.197)	0.003
Bacterial infection	4.113 (2.043–8.278)	<0.001		

p < 0.05 was considered significant.

### Plasma sCD36 was correlated with the prognosis of HBV-related liver diseases

**Figure 5:**
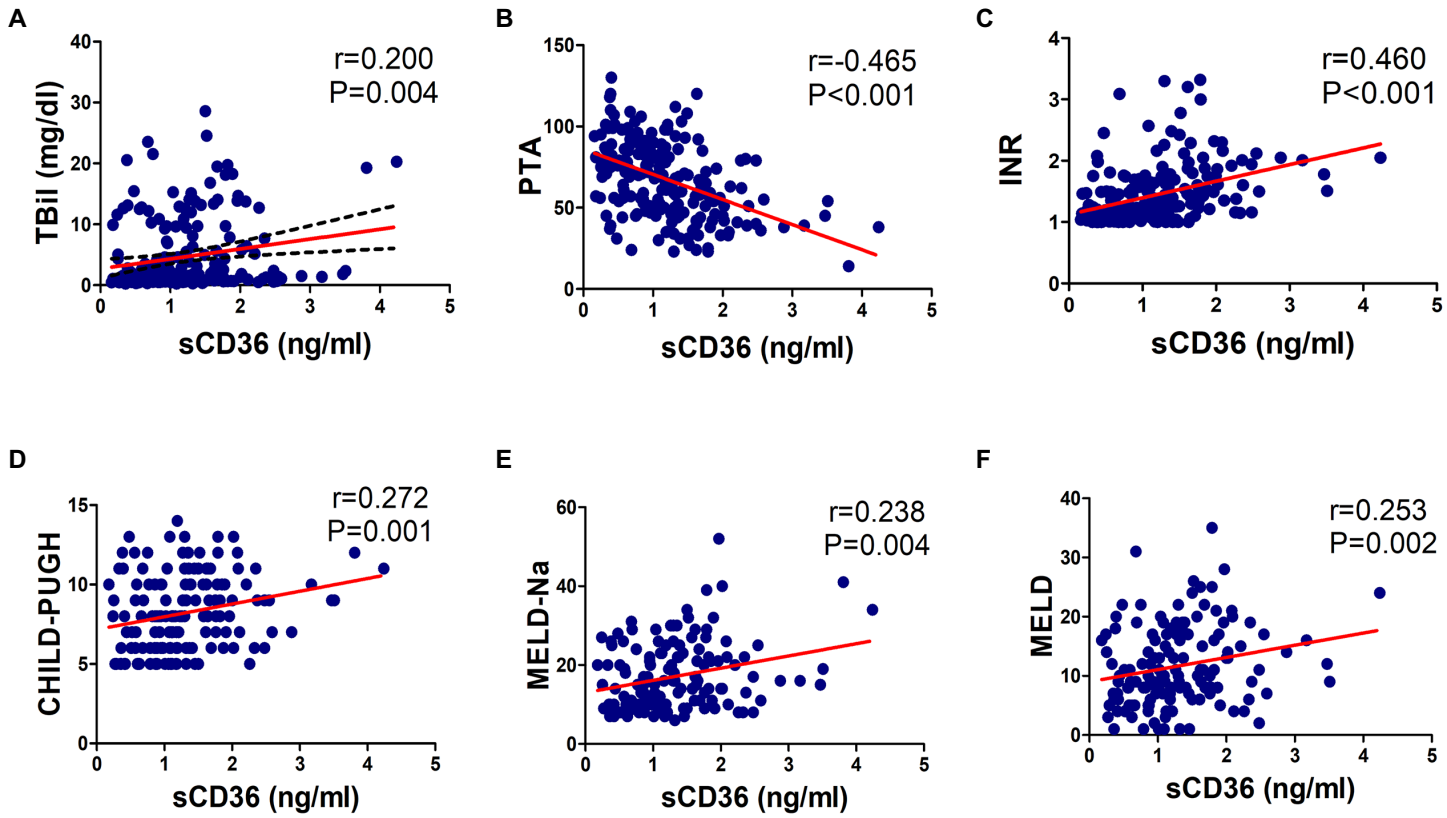
Correlation analysis between sCD36 and prognosis. In HBV-infected patients, the correlation between plasma sCD36 and **(A)** total bilirubin (TBil), **(B)** prothrombin activity (PTA), **(C)** international normalized ratio (INR). And in HBV-LC patients, the correlation between plasma sCD36 and **(D)** CHILD-PUGH score, **(E)** MELD-Na score and **(F)** MELD score. *p* values were calculated using the Spearman’s rank correlation for non-parametric data or Pearson’s correlation for parametric data.

Then, we explored whether CD36 could serve as a prognostic indicator for HBV-infected patients. As PTA, INR and TBil were common markers for assessing prognosis in HBV-related liver diseases, the correlation between plasma sCD36 and these markers (N = 205) were therefore analyzed. We found there was a significant negative correlation between sCD36 and PTA (*r* = −0.465, *p* < 0.05), and there exist positive correlation between sCD36 and serum TBil (*r* = 0.200, *p* < 0.05) or INR (*r* = 0.460, *p* < 0.05; Figures 5A–C). We then analyzed the correlation between plasma sCD36 and prognosis scores in HBV-LC patients. The results showed plasma sCD36 level was significantly associated with the prognostic scores, including CHILD-PUGH (*r* = 0.272, *p* < 0.05), MELD-Na (*r* = 0.238, *p* < 0.05) and MELD (*r* = 0.253, *p* < 0.05) scores in these patients (Figures 5D–F).

### Correlation between plasma sCD36 levels and monocyte or platelet CD36 expression

**Figure 6:**
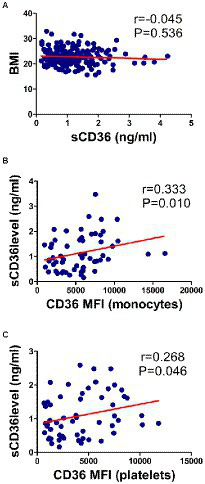
Correlation of sCD36 with body mass index (BMI), and CD36 expression on monocytes and platelets. **(A)** Correlation between sCD36 and BMI. **(B)** Correlation between sCD36 and CD36 expression on monocytes. **(C)** Correlation between sCD36 and CD36 expression on platelets. p values were calculated using the Spearman’s rank correlation.

We also attempted to explain the source of plasma sCD36 in this study. In HBV-infected patients (N = 205), the correlation between plasma sCD36 and BMI was analyzed. The mean BMI and sCD36 levels were 22.8 ± 3.2 kg/m2, 1.1 ± 0.7 ng/ml, respectively, and no significant correlation between plasma sCD36 and BMI in these patients was found (*r* = −0.045, *p* > 0.05; Figure 6A). We then analyzed the correlation between plasma sCD36 and monocyte or platelet CD36 expression in HBV-infected patients (N = 59). We found there was a moderate correlation between plasma sCD36 and monocyte CD36 expression (*r* = 0.333, *p* < 0.05), and a weak correlation between sCD36 and platelet CD36 expression (*r* = 0.268, *p* < 0.05; Figures 6B,C). These results suggested that sCD36 may be partially derived from the shedding of CD36 protein on the surface of platelets and monocytes.

## Discussion

CD36, identified as a class B scavenger receptor, also acts as a fatty acid transporter, therefore, it participated in both lipid metabolism and inflammation. The role of CD36 in liver disease has been recognized recently. CD36 is a central regulator in the development of nonalcoholic fatty liver disease (Zhao et al., 2018), and meanwhile, it plays an important role in the progression of liver cancer (Li et al., 2018). Our previous *in vitro* study has suggested CD36 was a positive regulator of HBV replication (Huang et al., 2017). Then, in this study we further investigated the clinical significance of CD36 in the progress of HBV-related liver diseases. We found a step-wise increase of sCD36 with the progression of chronic HBV infection from chronic hepatitis to cirrhosis and ultimately to liver failure, and we demonstrated sCD36 could serve as a noninvasive biomarker for predicting liver failure and prognosis in patients with HBV infection.

Chronic HBV infection is one of the most common chronic liver diseases with high morbidity and mortality. Accurate prediction for progression and prognosis of patients with chronic HBV infection is very important to choose appropriate management strategies, and detecting novel serum biomarkers associated with HBV-related liver diseases is critical to develop new diagnostic strategies. In this study, it was found that plasma sCD36 gradually increased with the severity of HBV-infected liver disease, and it could be used to predict liver failure in patients with liver cirrhosis. Firstly, we compared the differences of plasma sCD36 levels among different chronic HBV infected stages. It showed that the sCD36 levels were increased with the development of CHB to cirrhosis, and ultimately to liver failure. Secondly, correlation analysis demonstrated sCD36 levels were not related to serum HBV DNA load, but correlated with liver function parameters. Specifically, plasma sCD36 level was negatively correlated with serum albumin, platelets, and PTA, and positively related to total TBil. Thirdly, the ROC curve analysis indicated plasma sCD36 can be used to distinguish liver failure in patients with liver cirrhosis. And upon multivariate analysis, it was found that elevated sCD36 could serve as an effective independent predictor for liver failure in HBV-LC patients. Finally, we confirmed a correlation between sCD36 and MELD, MELD-Na, CHILD-PUGH scores in patients with HBV-LC. Altogether, we discovered there was a correlation between circulating sCD36 and the severity of HBV-related liver diseases, and sCD36 could be used to predict a poor prognosis for patients with HBV-LC.

As CD36 expression on platelets and monocytes contribute to a significant increase of sCD36 in many diseases and are involved in disease progression (Ockenhouse et al., 1989; Sampson et al., 2003; Liani et al., 2012), we also examined the expression of CD36 on platelets or monocytes. We found CD36 expression on platelets and monocytes in patients with HBV-LC were increased in comparison with that in CHB and healthy patients. However, platelets and monocyte CD36 expression were lower in HBV-LC patients with liver failure compared with those without. Although CD36 expression on platelets or monocytes exhibited significant correlations with some laboratory indices, such as albumin, serum cholinesterase, platelets and PTA, no significant correlations were found between platelets or monocyte CD36 expression and MELD, MELD-Na, CHILD-PUGH scores (data not shown). These results suggested that CD36 on platelets or monocytes may be partly involved in the development of HBV infection, but they are not ideal indicators for liver failure or prognosis. However, our results may be limited by sample size, and it is necessary to be verified in further studies.

Available knowledge suggests that sCD36 circulates in microvesicles in blood (Alkhatatbeh et al., 2011) and mainly sheds from platelets in healthy subjects (Gustafson et al., 2015). However, the correlation of sCD36 with CD36 expression on platelets was relatively weak. Notably, while the platelet counts in the HBV-LC (LF) group was the lowest, the sCD36 concentration was the highest among all groups, which implies the platelet CD36 is not the main source of plasma sCD36, especially in the HBV-LC (LF) group. Although a moderate correlation was observed between sCD36 and monocyte CD36 expression, CD36 expression on monocytes was not remarkably related to liver disease severity. Therefore, other cells may also contribute to the increase of sCD36 in circulation under pathological conditions. Several lines of evidence also demonstrated a significant positive correlation between sCD36 levels and hepatic CD36 mRNA and protein expression in NAFLD (Heeboll et al., 2017), suggesting circulating sCD36 may be derived from hepatocytes. In fact, it was found HBX protein can induce gene expression of CD36 in liver cells (Kim et al., 2007), and CD36 expression in HBV-replicating liver cell line is higher compared with original cell lines (Huang et al., 2016). On the other hand, there is evidence verifying that sCD36 levels are associated with CD36 expression on Kupffer cells in hepatitis C virus-related chronic liver diseases (Himoto et al., 2013). The Kupffer cells were gradually activated with progression of chronic HBV infection (Kazankov et al., 2014; Gronbaek et al., 2016). And activation of macrophage was shown to upregulate CD36 expression *in vitro* (Chavez-Sanchez et al., 2014; Zhang et al., 2015; Liang et al., 2016). Furthermore, in hyperlipidemia, it was demonstrated that multiple cell types produced sCD36, particularly with a strong contribution from endothelial cells (Biswas et al., 2021). Therefore, we speculated that a considerable source of plasma sCD36 might be derived from hepatocytes, Kupffer cells and endothelial cells. Altogether, it could be speculated that circulating sCD36 might be derived from multiple cell types in HBV-infected patients in this study. Further studies are required to clarify the sources of sCD36, as well as to elucidate the mechanisms of CD36 involved in HBV-related liver diseases.

Recently, noninvasive liver disease assessments in CHB patients have significantly progressed as several indices, scores or models derived from certain serum biomarkers or their combinations were brought up (Wu and Mao, 2020). These noninvasive indicators, including APRI, FIB-4, homocysteine (Hcy) and others, were proposed to be used in HBV-related liver diseases, such as CHB, HBV-ACLF and HBV-LC. For instance, APRI and FIB-4 were reported to predict liver fibrosis with a high accuracy in CHB patients (Li J. et al., 2015). Different from APRI or FIB-4, sCD36 was proved to be able to predict the liver failure and prognosis for patients with HBV-LC. Therefore, sCD36 as a biomarker had enriched the noninvasive liver disease assessments. On the other hand, as the results revealed sCD36 level did not corrected with serum HBV DNA load, but corrected with some liver function parameters, sCD36 probably work resemble Hcy, which might alter with liver damage since liver played an important role in its synthesis and metabolism (Ventura et al., 2005). And this could also explain why sCD36 level did not corrected with serum HBV DNA load. In addition, the weak correlation between sCD36 level with monocyte/platelet CD36 expression implied a considerable source of plasma sCD36 might be derived from other cell types, such as hepatocytes, Kupffer cells and endothelial cells. Thus, it was speculated that the plasma sCD36 level may represent the faulty of liver rather than the infection degree of HBV. Whereas, this speculation required to be further confirmed by elaborated investigation, which would be our next step after this work. Afterall, there are several advantages of sCD36 as a biomarker for HBV-related disease, which include: firstly, it is noninvasive, which makes the diagnosis easier and low-cost; secondly, the plasma level of sCD36 would help diagnosing different stage of HBV-related liver diseases, especially for predicting liver failure in HBV-related liver cirrhosis patients; thirdly, plasma sCD36 level could be used to predict the prognosis for HBV-infected patients.

In conclusion, we found a step-wise increase of sCD36 with the progression of chronic HBV infection from chronic hepatitis to cirrhosis and ultimately to liver failure, and plasma sCD36 could serve as a noninvasive biomarker for predicting liver failure and poor prognosis in patients with HBV infection. Therefore, plasma sCD36 may be combined with other biomarkers or techniques in clinic for prediction of HBV-related cirrhosis with liver failure in the future.

## Data availability statement

The original contributions presented in the study are included in the article/Supplementary material; further inquiries can be directed to the corresponding authors.

## Ethics statement

The studies involving human participants were reviewed and approved by Ethical Committee of the second affiliated hospital of Chongqing medical university (certificate no. 202011). The patients/participants provided their written informed consent to participate in this study. Written informed consent was obtained from the individual(s) for the publication of any potentially identifiable images or data included in this article.

## Author contributions

CC, AX, XL, EZ, YL, YL, and SZ performed the experiments. CC and AX analyzed the data, prepared figures, and contributed to the drafting of the manuscript. YC, PY, and ZT supervised this work and edited and revised manuscript. PY, ZT, and ZZ initiated the project, design the experiment, and approved the final version of manuscript. All authors contributed to the article and approved the submitted version.

## Funding

This research was supported by the National Natural Science Foundation of China (82202712), the General Program of Chongqing Natural Science Foundation (cstc2021jcyj-msxmX0061), the Chongqing Research Program of Basic Research and Frontier Technology (cstc2020jcyj-msxmX0205).

## Conflict of interest

The authors declare that the research was conducted in the absence of any commercial or financial relationships that could be construed as a potential conflict of interest.

## Publisher’s note

All claims expressed in this article are solely those of the authors and do not necessarily represent those of their affiliated organizations, or those of the publisher, the editors and the reviewers. Any product that may be evaluated in this article, or claim that may be made by its manufacturer, is not guaranteed or endorsed by the publisher.

## Supplementary material

The Supplementary material for this article can be found online at: https://www.frontiersin.org/articles/10.3389/fmicb.2022.1039614/full#supplementary-material

## Abbreviations

HBV: Hepatitis B virus
HC: Health control
CHB: Chronic hepatitis B
HBV-LC: Hepatitis B virus related liver cirrhosis
sCD36: Soluble CD36
APRI: Aspartate transaminase -platelet ratio index
FIB-4: Fibrosis 4 score
MELD: Model of end-stage liver disease
IL-6: Interleukin-6
TNF-α: tumor necrosis factor α
IL-8: Interleukin-8
NAFLD: Nonalcoholic fatty liver disease
HCV: Hepatitis C virus
HE: hepatic encephalopathy
GI: gastrointestinal
INR: international normalized ratio
PTA: Prothrombin activity
ACLF: acute-on-chronic liver failure
CLF: Chronic liver failure
HBV-LC (LF): Hepatitis B virus related liver cirrhosis with liver failure
HBV-LC (NO LF): Hepatitis B virus related liver cirrhosis without liver failure
ELISA: enzyme linked immunosorbent assay
APC: Allophycocyanin
PE: Allophycocyanin
ROC curve: Receiver operating characteristic curve
BMI: Body mass index
TBil: Total bilirubin
ALT: Alanine transaminase
AST: Aspartic transaminase
PLT: Platelet
Cr: creatinine
IQR: Interquartile range
OR: odds ratio
CI: Confidence interval
