# Supplementary material for: Circulating soluble CD36 as a novel biomarker for progression and prognosis of HBV-related liver diseases
Source: Front Microbiol. 2022 Nov 2;13:1039614. doi: 10.3389/fmicb.2022.1039614 (PMC9667018; doi:10.3389/fmicb.2022.1039614)
Supplement: Supplementary file 1 [file Data_Sheet_1.docx]

Supplementary Material

# Supplementary Data

Supplementary Material should be uploaded separately on submission. Please include any supplementary data, figures and/or tables. All supplementary files are deposited to FigShare for permanent storage and receive a DOI.

Supplementary material is not typeset so please ensure that all information is clearly presented, the appropriate caption is included in the file and not in the manuscript, and that the style conforms to the rest of the article. To avoid discrepancies between the published article and the supplementary material, please do not add the title, author list, affiliations or correspondence in the supplementary files.

# Supplementary Figures and Tables

For more information on Supplementary Material and for details on the different file types accepted, please see [here](http://home.frontiersin.org/about/author-guidelines#SupplementaryMaterial). Figures, tables, and images will be published under a Creative Commons CC-BY licence and permission must be obtained for use of copyrighted material from other sources (including re-published/adapted/modified/partial figures and images from the internet). It is the responsibility of the authors to acquire the licenses, to follow any citation instructions requested by third-party rights holders, and cover any supplementary charges.

## Supplementary Figures


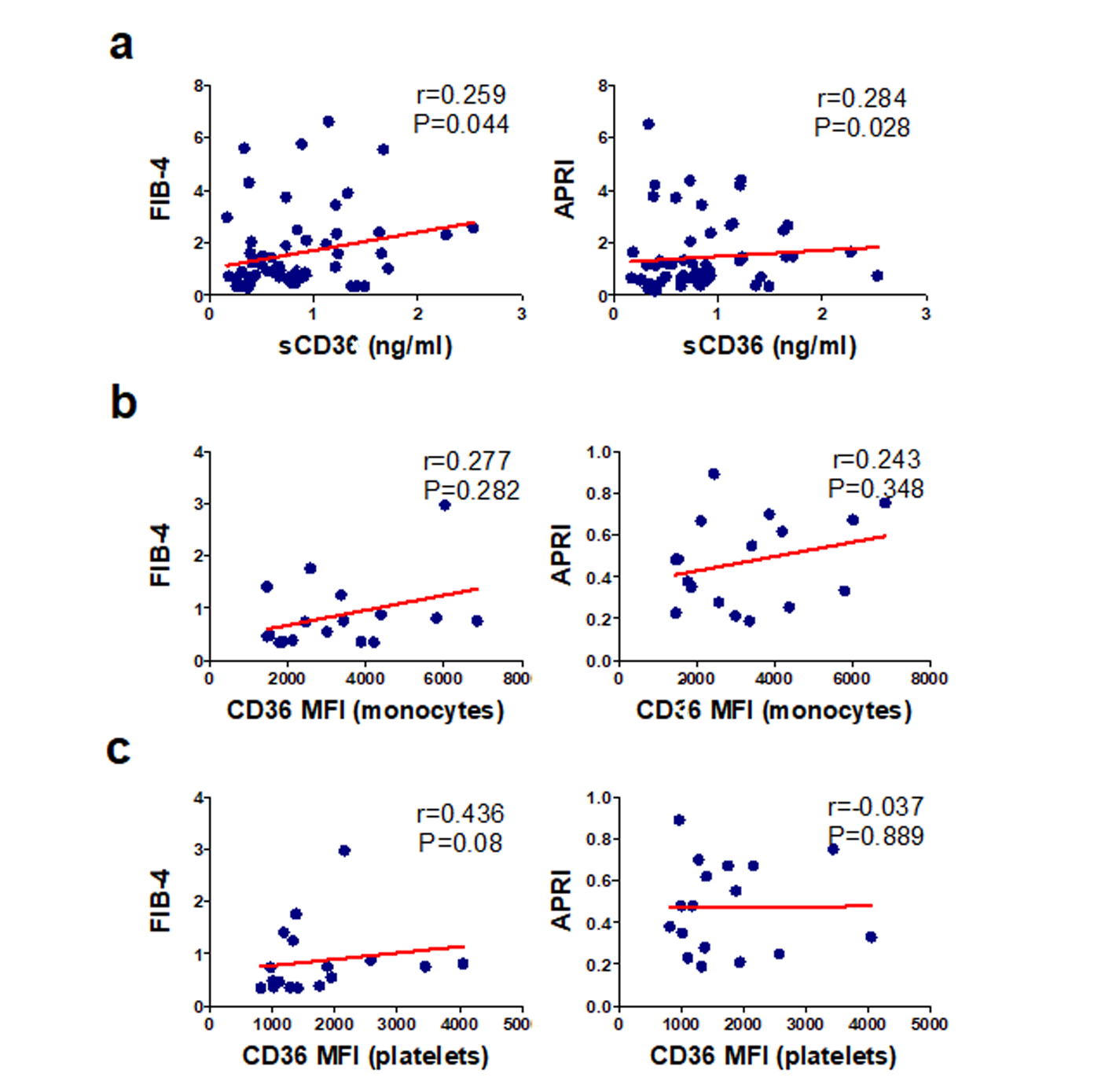


**Supplementary Figure 1.** **The correlation analysis between circulating CD36 expression and fibrosis scores in CHB patients.** (a) Correlation between sCD36 levels and the fibrosis markers, APRI and FIB-4. (b) Correlation between the expression of CD36 on monocytes and fibrosis markers, APRI and FIB-4. (c) Correlation between expression of CD36 on platelets and the fibrosis markers, APRI and FIB-4. P values were calculated using the Spearman's rank correlation. *P< 0.05, **P< 0.01, **P<0.001, ns, no statistical difference.

## Supplementary Tables

**Supplemental table 1. The correlation between plasma sCD36 level and clinical indexes in HBV-infected patients**

| **Parameter** | **sCD36<1ng/ml** | **sCD36≧1ng/ml** | **Pearson χ^2^** | **P value** |
| --- | --- | --- | --- | --- |
| Age (years) |  |  | 2.897 | 0.089 |
| ﹤50 | 66 | 61 |  |  |
| ≧50 | 31 | 47 |  |  |
| sex |  |  | 0.212 | 0.645 |
| Female | 27 | 27 |  |  |
| Male | 70 | 81 |  |  |
| PLT (*10^9^/L) |  |  | 15.408 | <0.001 |
| ﹤100 | 40 | 74 |  |  |
| ≧100 | 57 | 34 |  |  |
| TBil (mg/dl) |  |  | 5.447 | 0.020 |
| ﹤2 | 65 | 55 |  |  |
| ≧2 | 32 | 53 |  |  |
| ALT (U/L) |  |  | 3.597 | 0.058 |
| ﹤100 | 54 | 74 |  |  |
| ≧100 | 43 | 34 |  |  |
| AST (U/L) |  |  | 0.234 | 0.629 |
| ﹤100 | 68 | 79 |  |  |
| ≧100 | 29 | 29 |  |  |
| Albumin (g/L) |  |  | 13.230 | <0.001 |
| ﹤35 | 42 | 74 |  |  |
| ≧35 | 55 | 34 |  |  |
| INR |  |  | 36.073 | <0.001 |
| ≦1.3 | 73 | 36 |  |  |
| ＞1.3 | 24 | 72 |  |  |
| PTA |  |  | 21.210 | <0.001 |
| ﹤60 | 24 | 61 |  |  |
| ≧60 | 73 | 47 |  |  |
| Ascites |  |  | 8.508 | 0.004 |
| Yes | 25 | 49 |  |  |
| No | 72 | 59 |  |  |
| Bacterial infection |  |  | 12.441 | <0.001 |
| Yes | 26 | 55 |  |  |
| No | 71 | 53 |  |  |

P<0.05 was considered significant.
